# Antenatal Corticosteroid Administration in Late Preterm Singleton Pregnancies: A Propensity Score-weighted Analysis of Neonatal Outcomes

**DOI:** 10.1055/a-2869-5471

**Published:** 2026-05-18

**Authors:** Gulsah A. Yildiz, Goksu Goc, Hasan Sut, Necati B. Kaplan, Batuhan Aslan, Emel Okulu

**Affiliations:** 1 324617 Department of Obstetrics and Gynecology, Ankara University School of Medicine, AnkaraTürkiye; 2 666829 Department of Obstetrics and Gynecology, American Hospital, PrishtineKosovo; 3 666829 Department of Obstetrics and Gynecology, Spitalit Amerikan, PrishtinaKosovo; 4 324695 Department of Neonatology, Ankara University School of Medicine, AnkaraTürkiye

**Keywords:** late preterm birth, propensity score, respiratory morbidity, neonatal outcomes, antenatal corticosteroids, inverse probability

## Abstract

**Objective:**

This study aimed to evaluate the association between antenatal corticosteroid (ACS) exposure in late preterm singleton pregnancies (34 ^0/7^ –36 ^6/7^ weeks) and neonatal outcomes using propensity score-weighted analysis.

**Study Design:**

Retrospective single-center cohort of 1,012 singleton late preterm deliveries (2017–2022), excluding 75 pregnancies with ACS exposure before 34 weeks. Late-preterm exposure occurred in 126 pregnancies; 886 were unexposed. Stabilized inverse probability of treatment weighting, trimmed at the 1st and 99th percentiles, used prespecified maternal, obstetric, delivery, and fetal covariates. The primary outcome was documented initial respiratory support at birth.

**Results:**

Initial respiratory support occurred in 34.1% (43/126) of exposed neonates versus 25.2% (223/886) of unexposed neonates; weighted analysis did not show lower odds with exposure (odds ratio, 1.40; 95% confidence interval, 0.90–2.18; *p* = 0.130). Among exposed pregnancies, 85.7% received a single documented dose and 55.6% delivered within 24 hours. Neonatal hypoglycemia occurred in 3.2 versus 1.1% (odds ratio, 2.14; 95% confidence interval, 0.63–7.28; *P* = 0.224). No secondary outcomes remained significant after false discovery rate correction.

**Conclusion:**

In this real-world cohort with predominantly single-dose exposure and short ACS-to-delivery intervals, late preterm ACS was not associated with reduced initial respiratory support. These hypothesis-generating findings support selective rather than routine administration with individualized risk assessment and neonatal glucose monitoring.

**Key Points:**

- In a real-world cohort with predominantly single-dose exposure and short ACS-to-delivery intervals, late-preterm ACS exposure was not associated with reduced initial respiratory support at birth after propensity score weighting.
- The respiratory support signal was driven mainly by low-intensity hood oxygen, whereas higher-intensity support and hospitalization course duration outcomes did not show clinically clear benefit.
- Findings are hypothesis-generating and support selective rather than routine administration, with attention to ACS timing, course completion when feasible, and neonatal glucose monitoring.

## Introduction

Antenatal corticosteroid (ACS) administration to women at risk of preterm delivery is one of the most effective interventions in perinatal medicine, reducing neonatal respiratory distress syndrome, intraventricular hemorrhage, and mortality in births before 34 weeks of gestation. 1 2 3 The biological mechanism involves accelerated fetal lung maturation through enhanced surfactant production and structural lung development. 4 5 However, the benefit–harm balance in late preterm pregnancies (34 ^0/7^ to 36 ^6/7^ weeks) remains controversial. 6 7

The Antenatal Late Preterm Steroids (ALPS) trial demonstrated that betamethasone between 34 ^0/7^ and 36 ^5/7^ weeks reduced respiratory complications (relative risk = 0.80, 95% confidence interval [CI]: 0.66–0.97) but increased neonatal hypoglycemia requiring treatment (relative risk = 1.60, 95% CI: 1.37–1.87). 8 Subsequent meta-analyses confirmed respiratory benefits while highlighting concerns about metabolic harms and lack of long-term neurodevelopmental data. 9 10 However, later evidence has been less uniform. Yenuberi et al found no reduction in neonatal respiratory treatment need with late-preterm betamethasone, and the WHO ACTION trial did not reduce neonatal death or severe neonatal respiratory distress with late-preterm dexamethasone in a lower-resource setting. 11 12 Vieira et al found that full-course betamethasone in singleton gestations was associated with reduced respiratory complications but increased hypoglycemia, whereas partial-course findings were weaker. 13 A 2025 meta-analysis of randomized trials restricted to singleton late-preterm pregnancies reported no reduction in respiratory distress syndrome, although CPAP use for at least 2 hours and surfactant use were reduced. 14

After publication of the ALPS trial and subsequent professional guidance, late preterm ACS use increased in population datasets, yet implementation remains selective and heterogeneous across clinical contexts. 15 16 This reflects ongoing uncertainty about the benefit–harm balance in routine practice, including eligibility criteria, likelihood of birth within 7 days, previous corticosteroid exposure, diabetes, and local neonatal care resources. 17 Observational studies have yielded mixed results, highlighting the challenge of confounding by indication whereby clinicians selectively administer interventions to higher-risk pregnancies. 13 18 19 20

Target-trial emulation concepts can clarify how observational analyses approximate randomized evidence, and propensity score methods, particularly inverse probability of treatment weighting (IPTW), can reduce measured confounding by explicitly modeling treatment assignment. 21 22 23 IPTW preserves the entire sample and provides transparent diagnostics for assessing covariate balance, positivity, and weight stability. 24 25

We conducted a retrospective cohort study using rigorous propensity score-weighted analysis to evaluate the association between ACS exposure in the late preterm window and neonatal outcomes ( Supplementary Methods , available in the online version). We hypothesized that after comprehensive adjustment for measured confounding, late preterm ACS would not be associated with reduced initial neonatal respiratory support in routine practice. Secondary objectives included assessing potential metabolic harms and providing a transparent analytical workflow for future observational studies.

## Materials and Methods

### Study Design and Population

This retrospective cohort study included all singleton late preterm deliveries (34 + 0–36 + 6 weeks) at Ankara University School of Medicine between January 1, 2017 and December 31, 2022. The study was approved by the Ankara University School of Medicine Institutional Review Board (Protocol Number: I05-307-22, Date: May 27, 2022). Informed consent was waived due to the retrospective nature of the study.

Of 1,091 records screened, 1,087 were singleton late preterm deliveries. We excluded 75 pregnancies that received ACS before 34 weeks, leaving 1,012 deliveries (126 exposed to ACS in the late preterm window; 886 unexposed) for analysis ( Fig. 1 ).

**Fig. 1:**
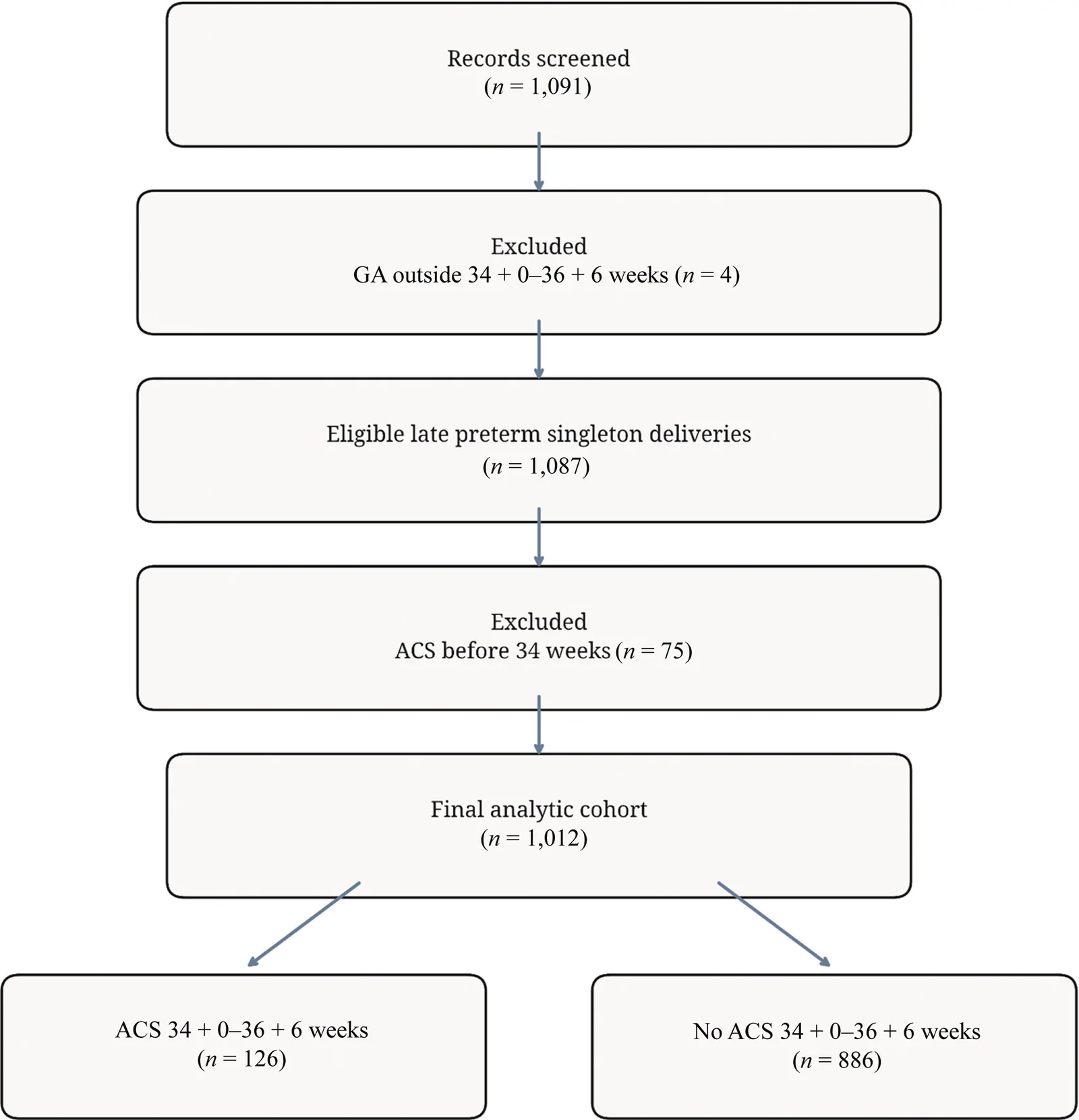
Study flow diagram. Of 1,091 records screened, 1,087 were singleton late preterm deliveries (34 ^0/7^ –36 ^6/7^ weeks). We excluded 75 pregnancies that received antenatal corticosteroids before 34 weeks, leaving 1,012 deliveries (126 exposed; 886 unexposed). ACS, antenatal corticosteroids; GA, gestational age.

### Exposure Definition

ACS exposure was defined as the administration of at least one dose of betamethasone or dexamethasone during the late preterm window (34 ^0/7^ –36 ^6/7^ weeks). Complete courses were two doses of betamethasone 12 mg intramuscularly 24 hours apart, or four doses of dexamethasone 6 mg intramuscularly 12 hours apart, consistent with standard protocols. 26 Incomplete courses were included in the exposed group using an intention-to-treat approach. Pregnancies that received ACS prior to 34 weeks were excluded to isolate late preterm exposure. The source dataset encoded the number of documented late preterm ACS doses and the ACS-to-delivery interval; these variables were summarized descriptively for exposed pregnancies.

### Clinical Practice and Treatment Assignment

During the study period, late preterm ACS administration at our center was not mandated by a universal protocol for all pregnancies between 34 ^0/7^ and 36 ^6/7^ weeks. Treatment was determined by the attending obstetric/perinatology team, generally for patients judged to have a meaningful likelihood of delivery within 7 days because of spontaneous preterm labor, premature rupture of membranes, hypertensive disease, fetal compromise, placental indications, or planned or anticipated medically indicated delivery. Previous ACS exposure, maternal infection, diabetes, and urgency of delivery were considered in clinical decision-making. Other management choices, including timing and mode of delivery, antibiotic use in premature rupture of membranes, and active versus expectant management, were made according to clinical indication and attending team judgment rather than study-driven protocols. This practice variation motivated the use of propensity score weighting and remains a potential source of residual confounding.

### Outcome Definitions

The primary outcome was documented initial respiratory support at birth/early neonatal stabilization, derived from the neonatal resuscitation and oxygen-support fields. The composite included hood oxygen, continuous positive airway pressure (CPAP), positive-pressure ventilation (PPV; coded as PBV in the source dataset), or intubation. Hospital-course respiratory support was evaluated separately using oxygen days, nasal/non-invasive mechanical ventilation days, invasive mechanical ventilation days, and NICU length of stay.

Secondary outcomes included neonatal intensive care unit (NICU) admission, neonatal hypoglycemia (blood glucose <40 mg/dL requiring intravenous dextrose), 27 neonatal sepsis (culture-proven or clinically treated infection within 72 hours), 28 pulmonary morbidity (respiratory distress syndrome, transient tachypnea of the newborn, or pneumonia), preterm complications (composite of necrotizing enterocolitis, intraventricular hemorrhage, or retinopathy of prematurity), and neonatal death (death within 28 days of birth). Continuous outcomes included NICU length of stay (days), oxygen days, invasive mechanical ventilation days, nasal/non-invasive ventilation days, 1-minute Apgar score, and 5-minute Apgar score. Total postnatal hospital length of stay was not available.

### Propensity Score Estimation and Weighting

Propensity scores were estimated using logistic regression with ACS exposure as the dependent variable. Covariates were selected a priori based on clinical knowledge, literature review, and directed acyclic graph construction 29 and included: maternal age, body mass index, gravidity, parity, conception method (spontaneous vs. assisted reproductive technology), maternal comorbidity group (hypertensive disorders, diabetes, thyroid disease, asthma, cholestasis, other), medication use group (antihypertensives, thyroid medication, anticoagulant, insulin/oral antidiabetic, antiepileptic, cholestasis medication, other), maternal infection, indication for delivery (preterm labor, premature rupture of membranes, preeclampsia, fetal distress, placental anomaly, oligohydramnios, other), delivery mode (vaginal vs. cesarean), and fetal sex. 30 31 Gestational age at delivery was not included in the primary propensity-score model and was evaluated separately through completed-week stratified analyses.

Delivery mode was included because cesarean delivery and absence of labor are associated with neonatal respiratory morbidity; however, absence of labor and planned versus intrapartum cesarean status were not consistently available.

Stabilized inverse probability of treatment weights were calculated as the marginal probability of treatment divided by the propensity score for exposed pregnancies, and the marginal probability of no treatment divided by one minus the propensity score for unexposed pregnancies. 32 Weights were trimmed at the 1st and 99th percentiles to reduce the influence of extreme weights. 33 The distribution of propensity scores and the stabilized trimmed weights are shown in Supplementary Figs. S1 and S2 (available in the online version).

### Statistical Analysis

Covariate balance after weighting was assessed using absolute standardized mean differences, with values <0.10 indicating adequate balance. 34 For binary outcomes, weighted odds ratios with 95% confidence intervals were estimated using logistic regression with robust standard errors. For continuous outcomes, weighted mean differences with 95% confidence intervals were estimated using linear regression with robust standard errors. Benjamini–Hochberg false discovery rate correction was applied to secondary outcomes only, an approach that controls the false discovery rate while remaining less conservative than Bonferroni-style adjustment. 35 36

Exploratory subgroup analyses stratified by completed gestational age in weeks were performed to assess potential effect modification. Sensitivity analyses included doubly robust weighted outcome models that additionally adjusted for propensity score covariates, a sensitivity cohort that included pregnancies exposed to ACS before 34 weeks (adding early ACS as a covariate), and an additional sensitivity analysis excluding delivery mode from the propensity score. Missing data were minimal (<2%) and were handled using complete-case analysis. All tests were two-sided with a significance level of 0.05. Analyses used R version 4.5.2 (R Foundation for Statistical Computing).

## Results

### Study Population and Baseline Characteristics

Among 1,091 records screened, 1,087 were singleton late preterm deliveries (34 ^0/7^ –36 ^6/7^ weeks). We excluded 75 (6.9%) pregnancies that received ACS before 34 weeks, yielding a final analytic cohort of 1,012 deliveries; 126 (12.5%) received late preterm ACS, and 886 (87.5%) did not ( Fig. 1 ).

Baseline characteristics are shown in Table 1 . The overall cesarean delivery rate was 60.4% (611/1,012). Before weighting, exposed pregnancies had higher cesarean delivery rates (76.2% versus 58.1%; standardized mean difference 0.39), lower mean gestational age at delivery (35.5 versus 36.0 weeks; standardized mean difference 0.53), and different distributions of delivery indications. Exposed pregnancies were more likely to have premature rupture of membranes (19.0 vs. 14.1%) and placental anomalies (10.3 vs. 5.0%). Labor induction was not uniformly coded in the retrospective dataset and therefore could not be analyzed reliably. After inverse probability of treatment weighting, covariate balance across all prespecified variables improved substantially, including delivery mode, with all absolute standardized mean differences below 0.10 ( Fig. 2 ).

**Fig. 2:**
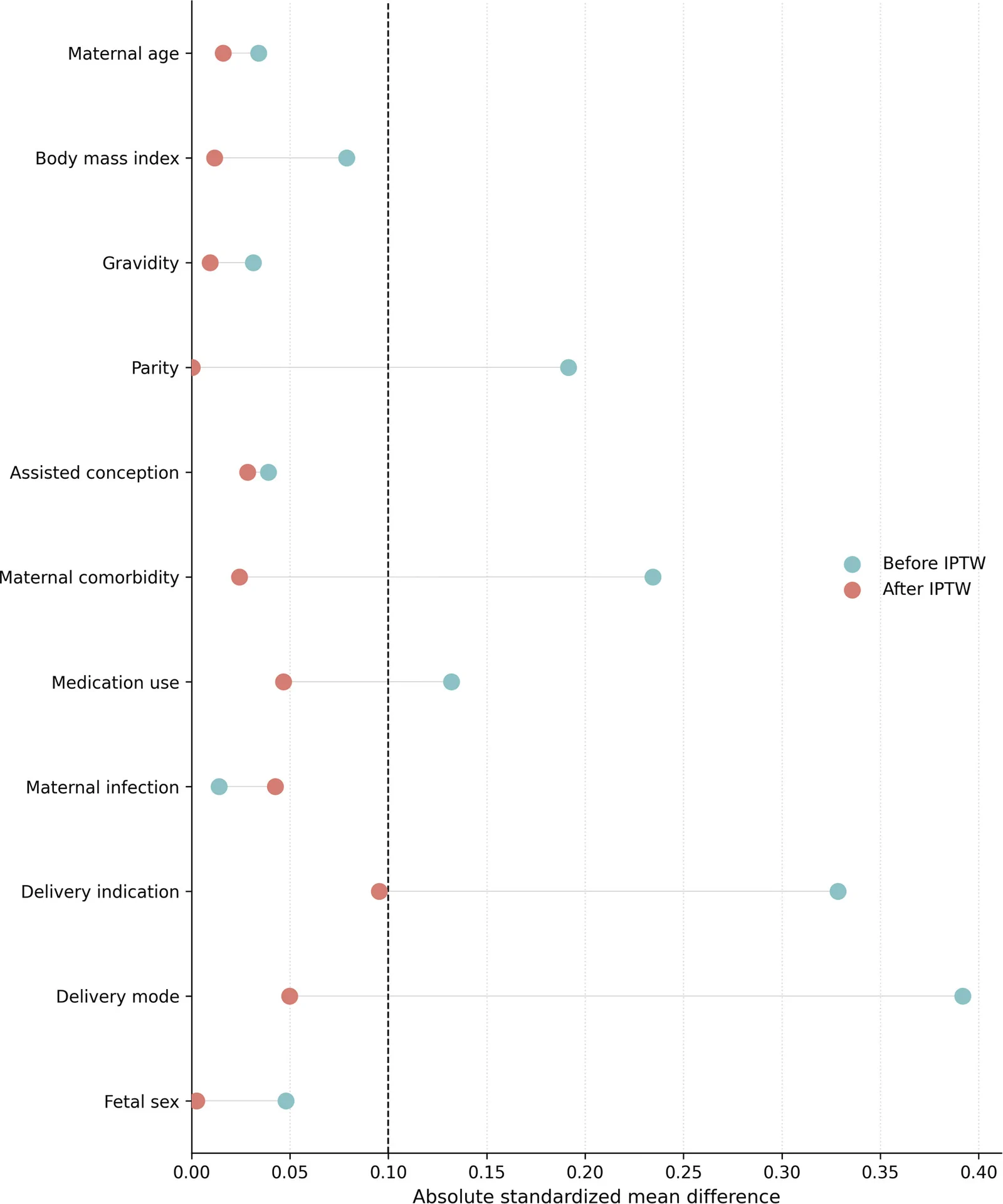
Covariate balance (Love plot). Absolute standardized differences for prespecified baseline covariates before and after inverse probability of treatment weighting (IPTW), demonstrating improved balance after weighting. Vertical dashed line at 0.10 represents adequate balance threshold. BMI, body mass index; IPTW, inverse probability of treatment weighting; SMD, standardized mean difference.

**Table 1:** Baseline characteristics (unweighted)

Characteristic	ACS ( *n* = 126)	No ACS ( *n* = 886)	SMD
Maternal age (y)	30.2 (5.1)	30.3 (5.7)	0.03
Body mass index (kg/m ^2^ )	29.8 (6.2)	29.3 (4.5)	0.08
Gravidity	2.3 (1.3)	2.4 (1.4)	0.03
Parity	0.8 (0.9)	1.0 (1.0)	0.19
GA at delivery (wk)	35.5 (0.8)	36.0 (0.8)	0.53
Conception method	–	–	0.04
Spontaneous	118 (93.7)	821 (92.7)	–
Assisted (IVF/IUI)	8 (6.3)	65 (7.3)	–
Type of delivery	–	–	0.39
Vaginal birth	30 (23.8)	371 (41.9)	–
Cesarean	96 (76.2)	515 (58.1)	–
Fetal sex	–	–	0.05
Female	57 (45.2)	422 (47.6)	–
Male	69 (54.8)	464 (52.4)	–
Maternal infection	–	–	0.01
No	122 (96.8)	860 (97.1)	–
Yes	4 (3.2)	26 (2.9)	–
Maternal comorbidity	–	–	0.25
None	78 (61.9)	612 (69.1)	–
Diabetes	8 (6.3)	62 (7.0)	–
Hypertensive disorders	13 (10.3)	34 (3.8)	–
Thyroid disease	10 (7.9)	75 (8.5)	–
Asthma	2 (1.6)	12 (1.4)	–
Cholestasis	1 (0.8)	8 (0.9)	–
Other	14 (11.1)	83 (9.4)	–
Medication use group	–	–	0.13
None	93 (73.8)	687 (77.5)	–
Thyroid medication	10 (7.9)	70 (7.9)	–
Anticoagulant	5 (4.0)	34 (3.8)	–
Antihypertensive	5 (4.0)	29 (3.3)	–
Insulin/oral antidiabetic	3 (2.4)	29 (3.3)	–
Antiepileptic	1 (0.8)	2 (0.2)	–
Cholestasis medication	1 (0.8)	4 (0.5)	–
Other	8 (6.3)	31 (3.5)	–
Indication for delivery	–	–	0.32
Preterm labor	65 (51.6)	598 (67.5)	–
PROM	24 (19.0)	125 (14.1)	–
Preeclampsia	9 (7.1)	42 (4.7)	–
Fetal distress	8 (6.3)	48 (5.4)	–
Placental anomaly	13 (10.3)	44 (5.0)	–
Oligohydramnios	2 (1.6)	7 (0.8)	–
Other/unknown	5 (4.0)	22 (2.5)	–

Abbreviations: ACS, antenatal corticosteroids; GA, gestational age; IUI, intrauterine insemination; IVF, in vitro fertilization; PROM, premature rupture of membranes; SMD, standardized mean difference.

Note: Values are mean (SD) or *n* (%). SMD is the absolute standardized mean difference; for categorical variables, SMD reflects the maximum absolute standardized difference across levels.

### Exposure Completeness and Timing

Among ACS-exposed pregnancies, 108 of 126 (85.7%) received a single documented dose and 18 (14.3%) received a documented double-dose course. Delivery occurred within 24 hours of ACS administration in 70 exposed pregnancies (55.6%), between 24 hours and 7 days in 48 (38.1%), and after more than 7 days in 8 (6.3%). Because the complete-course subgroup was small, dose-specific analyses were considered exploratory and imprecise ( Supplementary Table S1 , available in the online version); published evidence has shown that partial and complete ACS courses may produce different neonatal outcomes. 37

### Primary Outcome

Initial respiratory support at birth occurred in 34.1% (43/126) of exposed neonates compared with 25.2% (223/886) of unexposed neonates (crude OR = 1.54, 95% CI: 1.03–2.29, p = 0.034). In the propensity score-weighted analysis, late preterm corticosteroid exposure was not associated with lower odds of initial respiratory support at birth (IPTW OR = 1.40, 95% CI: 0.90–2.18, *p* = 0.130; Table 2 ).

**Table 2:** Primary and secondary neonatal outcomes

Outcome	ACS *n* / *N* (%)	No ACS *n* / *N* (%)	Crude OR (95% CI)	IPTW OR (95% CI)	*p* -Value	*q* -Value
Primary outcome						
Respiratory support a	43/126 (34.1)	223/886 (25.2)	1.54 (1.03–2.29)	1.40 (0.90–2.18)	0.130	—
Secondary outcomes						
NICU admission	35/126 (27.8)	204/886 (23.0)	1.29 (0.84–1.96)	1.10 (0.69–1.75)	0.685	0.723
Hypoglycemia	4/126 (3.2)	10/886 (1.1)	2.87 (0.89–9.30)	2.14 (0.63–7.28)	0.224	0.672
Neonatal sepsis	5/126 (4.0)	21/886 (2.4)	1.70 (0.63–4.60)	1.34 (0.47–3.83)	0.585	0.723
Pulmonary morbidity b	18/126 (14.3)	136/886 (15.3)	0.92 (0.54–1.56)	0.87 (0.48–1.56)	0.632	0.723
Preterm complications	5/126 (4.0)	18/886 (2.0)	1.99 (0.73–5.47)	2.44 (0.79–7.48)	0.120	0.672
Neonatal death	1/126 (0.8)	9/886 (1.0)	0.78 (0.10–6.21)	1.46 (0.18–11.63)	0.723	0.723

Abbreviations: ACS, antenatal corticosteroids; CI, confidence interval; IPTW, inverse probability of treatment weighting; NICU, neonatal intensive care unit; OR, odds ratio.

Note: *q* -values are Benjamini–Hochberg false discovery rate-adjusted *q* -values for secondary outcomes only.

a Primary endpoint: documented initial respiratory support at birth/early neonatal stabilization, derived from the neonatal resuscitation and oxygen-support category fields. It included hood oxygen, CPAP, PPV, or intubation.

b Secondary endpoint based on the respiratory morbidity category; interpret cautiously because diagnosis fields may have variable completeness.

Initial support consisted of hood oxygen only in 14 (11.1%), CPAP in 21 (16.7%), PPV in 6 (4.8%), and intubation in 2 (1.6%) exposed neonates. In the unexposed group, the corresponding rates were 34 (3.8%), 162 (18.3%), 22 (2.5%), and 5 (0.6%), respectively. Higher-intensity support, defined as CPAP, PPV, or intubation, occurred in 29 exposed neonates (23.0%) and 189 unexposed neonates (21.3%; Supplementary Table S2 , available in the online version).

### Secondary Outcomes

Neonatal hypoglycemia was uncommon (3.2% [4/126] vs. 1.1% [10/886]), and estimates were imprecise (IPTW OR = 2.14, 95% CI: 0.63–7.28, *P* = 0.224). NICU admission occurred in 27.8% (35/126) of exposed versus 23.0% (204/886) of unexposed neonates (IPTW OR = 1.10, 95% CI: 0.69–1.75, *P* = 0.685). Neonatal sepsis occurred in 4.0% (5/126) versus 2.4% (21/886; IPTW OR = 1.34, 95% CI: 0.47–3.83, *p* = 0.585). Pulmonary morbidity occurred in 14.3% (18/126) versus 15.3% (136/886; IPTW OR = 0.87, 95% CI: 0.48–1.56, *p* = 0.632). Preterm complications occurred in 4.0% (5/126) versus 2.0% (18/886; IPTW OR = 2.44, 95% CI: 0.79–7.48, *p* = 0.120). Neonatal death occurred in 0.8% (1/126) versus 1.0% (9/886; IPTW OR = 1.46, 95% CI: 0.18–11.63, *p* = 0.723). After false discovery rate correction, no secondary outcomes remained significant (all *q* -values >0.67; Table 2 ). Continuous neonatal outcomes are presented in Supplementary Table S3 (available in the online version).

In weighted linear models, there were no material differences in NICU length of stay (mean difference = 0.83 days, 95% CI: −0.49 to 2.15, *p* = 0.218), oxygen days (mean difference = 0.95 days, 95% CI: −0.09 to 1.99, *p* = 0.074), invasive mechanical ventilation days (mean difference = 0.37 days, 95% CI: −0.21 to 0.95, *p* = 0.213), nasal/non-invasive ventilation days (mean difference = 0.21 days, 95% CI: −0.12 to 0.53, *p* = 0.221), 1-minute Apgar score (mean difference = − 0.09, 95% CI: −0.46 to 0.29, *p* = 0.652), or 5-minute Apgar score (mean difference = 0.03, 95% CI: −0.24 to 0.31, *p* = 0.806).

### Gestational Age–stratified Analysis

Exploratory analyses stratified by completed gestational age week are presented in Table 3 . For the primary outcome of initial respiratory support at birth, IPTW estimates were imprecise: OR = 0.62 (95% CI: 0.25–1.53, *p* = 0.305) at 34 weeks, OR = 0.69 (95% CI: 0.29–1.62, *p* = 0.391) at 35 weeks, and OR = 2.25 (95% CI: 1.07–4.72, *p* = 0.033) at 36 weeks. These exploratory subgroup findings should be interpreted with caution due to small sample sizes, sparse events within strata, and the lack of adjustment for multiple subgroup comparisons.

**Table 3:** Exploratory gestational age-stratified IPTW analyses for selected outcomes

Outcome by GA week	IPTW OR	95% CI	*p* -Value
34 weeks ( *n* = 139)			
Initial respiratory support	0.62	0.25–1.53	0.305
NICU admission	0.46	0.19–1.13	0.092
Hypoglycemia	0.78	0.12–5.05	0.799
Neonatal sepsis	0.51	0.09–2.85	0.441
35 weeks ( *n* = 307)			
Initial respiratory support	0.69	0.29–1.62	0.391
NICU admission	0.54	0.22–1.35	0.190
Hypoglycemia	1.06	0.12–9.45	0.960
Neonatal sepsis	1.96	0.23–17.05	0.541
36 weeks ( *n* = 566)			
Initial respiratory support	2.25	1.07–4.72	0.033
NICU admission	1.11	0.42–2.89	0.837
Hypoglycemia	4.28	0.37–48.89	0.242
Neonatal sepsis	4.31	0.82–22.60	0.084

Abbreviations: CI, confidence interval; GA, gestational age; IPTW, inverse probability of treatment weighting; NICU, neonatal intensive care unit; OR, odds ratio.

Note: These exploratory analyses are stratified by completed gestational age week. Subgroup sample sizes are small and these results should be considered hypothesis-generating. IPTW models were fit separately within each gestational age stratum, and subgroup estimates were not adjusted for multiple subgroup comparisons.

### Sensitivity Analysis

A doubly robust weighted logistic regression (IPTW plus covariate adjustment) yielded similar findings for the primary outcome (OR = 1.41, 95% CI: 0.87–2.27, *p* = 0.159; Supplementary Table S4 , available in the online version). Results were also similar in a sensitivity cohort that included pregnancies exposed to ACS before 34 weeks (IPTW OR = 1.37, 95% CI: 0.89–2.09, *p* = 0.150). In an additional sensitivity analysis excluding delivery mode from the propensity score, the primary-outcome estimate was larger (IPTW OR = 1.54, 95% CI: 1.01–2.35, *p* = 0.046; doubly robust OR = 1.57, 95% CI: 1.02–2.42, *p* = 0.042), supporting delivery mode as an important measured confounder.

## Discussion

### Principal Findings

In this propensity score-weighted analysis of 1,012 singleton late preterm deliveries, ACS exposure between 34 and 36 weeks was not associated with reduced initial respiratory support at birth after comprehensive adjustment for measured confounding. The crude analysis suggested increased odds (OR = 1.54), but this was attenuated after weighting (OR = 1.40, 95% CI: 0.90–2.18). No secondary outcomes showed significant associations after multiple testing correction. Exploratory gestational age–stratified analyses were imprecise and did not demonstrate robust evidence of effect modification. Because exposure assignment was not randomized and several clinically important variables were unavailable, these estimates should be interpreted as adjusted associations and hypothesis-generating rather than definitive causal effects.

### Comparison with Existing Literature

Our findings contrast with the ALPS trial, which demonstrated a 20% relative risk reduction in a composite respiratory outcome within 72 hours after birth. 8 More recent randomized evidence has been mixed. Yenuberi et al reported no reduction in neonatal respiratory treatment need after late-preterm betamethasone, and the WHO ACTION trial did not reduce neonatal death, stillbirth or neonatal death, or severe neonatal respiratory distress after late-preterm dexamethasone in India. 11 12 A 2025 meta-analysis of randomized trials in singleton late-preterm pregnancies similarly found no reduction in respiratory distress syndrome, although some treatment endpoints, including CPAP use for at least 2 hours and surfactant use, were reduced. 14 Several factors may explain divergence across studies. First, the ALPS primary outcome included CPAP or high-flow nasal cannula for at least 2 hours, supplemental oxygen with fraction of inspired oxygen of at least 0.30 for at least 4 hours, mechanical ventilation, extracorporeal membrane oxygenation, stillbirth, or neonatal death, whereas our primary outcome was a recorded initial respiratory-support composite at birth/early stabilization. 8 Second, the ALPS trial was conducted under randomized conditions with standardized eligibility criteria, whereas our cohort reflects routine care, in which ACS was selectively administered to patients perceived to be at higher risk. Third, unmeasured factors such as indication severity, planned cesarean without labor, urgency of delivery, and local neonatal care thresholds may persist despite measured covariate balance.

Observational studies have also yielded mixed results. In a Mount Sinai retrospective cohort, Vieira et al reported that full-course betamethasone among singletons was associated with lower odds of respiratory complications but higher odds of hypoglycemia, whereas partial-course exposure was not significantly associated with respiratory benefit in singletons. 13 This finding is directly relevant to our cohort, in which only 18 exposed pregnancies (14.3%) had a documented double-dose course. A nationwide population-based study in Taiwan reported lower respiratory support but increased neonatal glucose instability after late preterm ACS exposure. 18 In a single-center cohort of growth-restricted late preterm pregnancies, ACS did not significantly decrease the need for respiratory support and was associated with increased hypoglycemia. 19 Another single-center study reported frequent suboptimal dosing and heterogeneous neonatal outcomes, emphasizing that real-world effectiveness may depend on timing, dose completion, indication, and local practice. 20 Although the IPTW odds ratio for the primary outcome remained greater than 1, this should not be interpreted as definitive evidence of harm because the confidence interval included both potential benefit and potential harm. Rather, the estimate likely reflects a combination of residual confounding by indication, incomplete or delayed exposure, and differences in early neonatal support thresholds.

Current professional guidance supports a selective rather than universal approach. ACOG and SMFM recommend or offer a single course of betamethasone for appropriately selected patients with singleton pregnancies between 34 ^0/7^ and 36 ^6/7^ weeks who are at high risk of delivery within 7 days and have not previously received ACS. 2 38 SMFM further recommends against late preterm ACS when the likelihood of delivery before 37 weeks is low and recommends against use in pregestational diabetes because of hypoglycemia concerns. 38 WHO recommendations differ in some respects and do not recommend ACS for planned cesarean delivery at 34 ^0/7^ to 36 ^6/7^ weeks. 39 Our findings align with this selective framework and underscore that local baseline risk, gestational age, mode of delivery, expected latency to delivery, and probability of course completion should inform treatment decisions.

### Neonatal Hypoglycemia

The potential increase in neonatal hypoglycemia (weighted odds ratio = 2.14, though not significant) is consistent with the ALPS trial and professional guidance emphasizing hypoglycemia concerns. 8 38 Corticosteroid exposure may impair neonatal glucose homeostasis, particularly in late preterm neonates with limited glycogen stores. 27 The ALPS trial reported a 60% increase in hypoglycemia requiring treatment. 8 Our point estimate was similar but with wider confidence intervals due to lower event rates.

### Gestational Age–Stratified Findings

Exploratory gestational age–stratified analyses were imprecise and should be considered hypothesis-generating. The estimates for initial respiratory support differed across completed gestational age weeks, but these results should not be interpreted as evidence that late preterm ACS is beneficial or harmful at a specific gestational age in this dataset because the analyses were sparse, exploratory, and not adjusted for multiple subgroup comparisons. Larger studies are needed to evaluate gestational age, planned delivery mode, labor status, ACS dose completion, and ACS-to-delivery interval together.

### Neurodevelopmental Considerations

An important dimension of the benefit–harm balance for late preterm ACS is its potential effect on long-term neurodevelopment. The ALPS follow-up study evaluated children at age 6 years and found no significant differences in neurodevelopmental outcomes between betamethasone and placebo groups (composite adverse outcome: 21.0 vs. 19.2%). 40 However, a large population-based cohort from Ontario, Canada, including 25,668 late preterm infants, reported that ACS exposure was associated with increased risk of suspected neurocognitive disorder (adjusted hazard ratio = 1.12, 95% CI: 1.05–1.20) and increased audiometry testing (adjusted hazard ratio 1.20, 95% confidence interval 1.10–1.31) by 5 years of age. 41 A recent systematic review and meta-analysis including 14 studies found that 19 of 23 neurodevelopmental outcomes showed no association with ACS administration, and among studies with low or moderate risk of bias, 14 of 15 outcomes showed no significant association. 42 Together, these data are reassuring but not definitive, and they support careful patient selection until longer-term evidence is more complete.

### Strengths and Limitations

Strengths include a prespecified propensity score-weighting strategy with explicit balance diagnostics, weight truncation to reduce the impact of extreme weights, and a doubly robust sensitivity analysis. We achieved excellent covariate balance (all standardized mean differences less than 0.10) with minimal precision loss. Sensitivity analyses using alternative methods yielded consistent results. We applied false discovery rate correction for multiple testing of secondary outcomes.

Limitations include the retrospective single-center design, potential residual confounding by unmeasured factors, and missingness for several neonatal morbidity outcomes (particularly pulmonary morbidity and ventilation duration), which reduced effective sample size and widened confidence intervals. Although delivery mode was included in the propensity score and was balanced after weighting, cesarean delivery remained a major clinical issue in this cohort, and the absence of labor, planned versus intrapartum cesarean delivery, and indication severity could not be fully captured. Compared with an ideal target trial, treatment timing, indication severity, labor status, and urgency of delivery could not be standardized or fully measured. 21 We were able to describe documented ACS dose completeness and ACS-to-delivery timing, but complete-course and latency-specific subgroup analyses were limited by small exposed-cell sizes and potential confounding by delivery urgency. Labor induction, total hospital length of stay, antibiotic administration, active versus expectant management of PROM, planned versus intrapartum cesarean, and labor status were not uniformly coded and therefore could not be incorporated. Finally, NICU admission and early respiratory-support decisions may be influenced by local admission thresholds, staffing, and neonatal care pathways, which may limit generalizability to other practice settings. Gestational age at delivery showed substantial unweighted imbalance between exposure groups (35.5 versus 36.2 weeks) but was not included in the primary propensity-score model because it lies on the causal pathway between exposure decision (at the time of suspected late preterm delivery) and the neonatal outcome; instead, completed-week stratified analyses were used to evaluate effect modification by gestational age.

### Clinical Implications

Our findings support a selective approach to late preterm ACS administration, with individualized risk assessment and careful consideration of potential benefits and harms. Patients are most biologically plausible to benefit when delivery before 37 weeks is likely, sufficient latency exists for fetal lung maturation, and baseline respiratory risk is high, such as earlier late preterm gestational age or planned cesarean delivery without labor. Expected benefit is less certain when delivery timing is unclear, birth is imminent within only a few hours, course completion is unlikely, prior ACS exposure exists, pregestational diabetes is present, or gestational age approaches 37 weeks. This approach is consistent with recent evidence suggesting that absolute respiratory benefit from late preterm betamethasone varies according to gestational age and planned mode of delivery. 43 When ACS is administered, enhanced neonatal glucose monitoring is essential, given consistent hypoglycemia signals. Protocols should include frequent glucose checks in the first 24 to 48 hours, with low intervention thresholds. 44

Future research should focus on identifying subgroups most likely to benefit from late preterm ACS, developing prediction models to guide individualized treatment decisions, and evaluating long-term neurodevelopmental and metabolic outcomes. Large-scale pragmatic trials or registry studies with long-term follow-up are needed to establish the benefit-harm balance in diverse populations. Given the limited and partly conflicting evidence on long-term neurodevelopmental outcomes, careful and selective application remains warranted until more definitive longitudinal data are available.

## Conclusion

In this single-center cohort of late preterm singleton deliveries, late preterm ACS exposure was not associated with reduced initial respiratory support at birth after IPTW adjustment. The cohort was characterized by predominantly single-dose exposure and short ACS-to-delivery intervals, emphasizing the challenge of translating trial efficacy into real-world effectiveness. Estimates for secondary outcomes were imprecise, underscoring the need for careful documentation and larger studies with improved measurement of indication severity, planned delivery mode and labor status, ACS dose completeness, and ACS-to-delivery timing. These hypothesis-generating findings support selective administration of late preterm ACS with individualized risk assessment and careful neonatal monitoring for hypoglycemia.

## References

[1] Roberts D Brown J Medley N Dalziel S R Antenatal corticosteroids for accelerating fetal lung maturation for women at risk of preterm birth Cochrane Database Syst Rev 2017 3 03 CD004454 10.1002/14651858.CD004454.pub3 PMC6464568 28321847

[2] Committee on Obstetric Practice Committee Opinion No. 713: antenatal corticosteroid therapy for fetal maturation Obstet Gynecol 2017 130 02 e102 e109 10.1097/AOG.0000000000002237 28742678

[3] Liggins G C Howie R N A controlled trial of antepartum glucocorticoid treatment for prevention of the respiratory distress syndrome in premature infants Pediatrics 1972 50 04 515 525 4561295

[4] Jobe A H Soll R F Choice and dose of corticosteroid for antenatal treatments Am J Obstet Gynecol 2004 190 04 878 881 10.1016/j.ajog.2004.01.044 15118606

[5] Ballard P L Ballard R A Scientific basis and therapeutic regimens for use of antenatal glucocorticoids Am J Obstet Gynecol 1995 173 01 254 262 10.1016/0002-9378(95)90210-4 7631700

[6] Saccone G Berghella V Antenatal corticosteroids for maturity of term or near term fetuses: systematic review and meta-analysis of randomized controlled trials BMJ 2016 355 i5044 10.1136/bmj.i5044 PMC5062056 27733360

[7] Melamed N Shah J Soraisham A Association between antenatal corticosteroid administration-to-birth interval and outcomes of preterm neonates Obstet Gynecol 2015 125 06 1377 1384 10.1097/AOG.0000000000000840 26000509

[8] Gyamfi-Bannerman C Thom E A Blackwell S C NICHD Maternal–Fetal Medicine Units Network Antenatal betamethasone for women at risk for late preterm delivery N Engl J Med 2016 374 14 1311 1320 10.1056/NEJMoa1516783 PMC4823164 26842679

[9] Saccone G Berghella V Maruotti G M ACS for women at risk of late preterm delivery: a systematic review and meta-analysis of randomized trials J Matern Fetal Neonatal Med 2016 29 14 2325 2330

[10] Deshmukh M Patole S Antenatal corticosteroids for impending late preterm (34-36+6 weeks) deliveries—a systematic review and meta-analysis of RCTs PLoS One 2021 16 03 e0248774 10.1371/journal.pone.0248774 PMC7984612 33750966

[11] Yenuberi H Ross B Sasmita Tirkey R Late-preterm antenatal steroids for reduction of neonatal respiratory complications: a randomized controlled trial Obstet Gynecol 2024 143 04 468 474 10.1097/AOG.0000000000005520 38330411

[12] WHO ACTION Trials Collaborators Antenatal dexamethasone for late preterm birth: a multi-centre, two-arm, parallel, double-blind, placebo-controlled, randomized trial EClinicalMedicine 2022 44 101285 10.1016/j.eclinm.2022.101285 PMC8850324 35198915

[13] Vieira L A Kao Y H Tsevdos D S Late preterm antenatal corticosteroids in singleton and twin gestations: a retrospective cohort study BMC Pregnancy Childbirth 2022 22 01 904 10.1186/s12884-022-05262-1 PMC9721054 36471280

[14] Zullo F Gulersen M Di Mascio D Antenatal corticosteroids for patients at risk of late preterm birth: a systematic review and meta-analysis of randomized controlled trials Am J Obstet Gynecol MFM 2025 7 08 101709 10.1016/j.ajogmf.2025.101709 40418984

[15] Kearsey E OR Been J V Souter V L Stock S J The impact of the antenatal late preterm steroids trial on the administration of antenatal corticosteroids Am J Obstet Gynecol 2022 227 02 2800 2.8E17 10.1016/j.ajog.2022.03.037 35341727

[16] Clapp M A Melamed A Freret T S James K E Gyamfi-Bannerman C Kaimal A J US incidence of late-preterm steroid use and associated neonatal respiratory morbidity after publication of the Antenatal Late Preterm Steroids trial, 2015–2017 JAMA Netw Open 2022 5 05 e2212702 10.1001/jamanetworkopen.2022.12702 PMC9118048 35583868

[17] Kamath-Rayne B D DeFranco E A Marcotte M P Antenatal steroids for treatment of fetal lung immaturity after 34 weeks of gestation: an evaluation of neonatal outcomes Obstet Gynecol 2012 119 05 909 916 10.1097/AOG.0b013e31824ea4b2 PMC3608417 22525901

[18] Liang F W Tsai H F Kuo P L Tsai P Y Antenatal corticosteroid therapy in late preterm delivery: a nationwide population-based retrospective study in Taiwan BJOG 2021 128 09 1497 1502 10.1111/1471-0528.16677 33629515

[19] Bitar G Merrill S J Sciscione A C Hoffman M K Antenatal corticosteroids in the late preterm period for growth-restricted pregnancies Am J Obstet Gynecol MFM 2020 2 03 100153 10.1016/j.ajogmf.2020.100153 33345883

[20] Go M Wahl M Kruss T McEvoy C T Late preterm antenatal steroid use and infant outcomes in a single center J Perinatol 2024 44 07 1009 1013 10.1038/s41372-024-01934-2 PMC12755485 38499754

[21] Hernán M A Robins J M Using big data to emulate a target trial when a randomized trial is not available Am J Epidemiol 2016 183 08 758 764 10.1093/aje/kwv254 PMC4832051 26994063

[22] Rosenbaum P R Rubin D B The central role of the propensity score in observational studies for causal effects Biometrika 1983 70 01 41 55

[23] Austin P C An introduction to propensity score methods for reducing the effects of confounding in observational studies Multivariate Behav Res 2011 46 03 399 424 10.1080/00273171.2011.568786 PMC3144483 21818162

[24] Austin P C Stuart E A Moving towards best practice when using inverse probability of treatment weighting (IPTW) using the propensity score to estimate causal treatment effects in observational studies Stat Med 2015 34 28 3661 3679 10.1002/sim.6607 PMC4626409 26238958

[25] Cole S R Hernán M A Constructing inverse probability weights for marginal structural models Am J Epidemiol 2008 168 06 656 664 10.1093/aje/kwn164 PMC2732954 18682488

[26] Brownfoot F C Gagliardi D I Bain E Middleton P Crowther C A Different corticosteroids and regimens for accelerating fetal lung maturation for women at risk of preterm birth Cochrane Database Syst Rev 2013 08 CD006764 10.1002/14651858.CD006764.pub3 23990333

[27] Adamkin D H Committee on Fetus and Newborn Postnatal glucose homeostasis in late-preterm and term infants Pediatrics 2011 127 03 575 579 10.1542/peds.2010-3851 21357346

[28] Puopolo K M Benitz W E Zaoutis T E Committee on Fetus and Newborn Committee on Infectious Diseases Management of neonates born at ≥35 0/7 weeks' gestation with suspected or proven early-onset bacterial sepsis Pediatrics 2018 142 06 e20182894 10.1542/peds.2018-2894 30455342

[29] Greenland S Pearl J Robins J M Causal diagrams for epidemiologic research Epidemiology 1999 10 01 37 48 9888278

[30] Hernán M A Hernández-Díaz S Robins J M A structural approach to selection bias Epidemiology 2004 15 05 615 625 10.1097/01.ede.0000135174.63482.43 15308962

[31] VanderWeele T J Principles of confounder selection Eur J Epidemiol 2019 34 03 211 219 10.1007/s10654-019-00494-6 PMC6447501 30840181

[32] Robins J M Hernán M A Brumback B Marginal structural models and causal inference in epidemiology Epidemiology 2000 11 05 550 560 10.1097/00001648-200009000-00011 10955408

[33] Lee B K Lessler J Stuart E A Weight trimming and propensity score weighting PLoS One 2011 6 03 e18174 10.1371/journal.pone.0018174 PMC3069059 21483818

[34] Austin P C Balance diagnostics for comparing the distribution of baseline covariates between treatment groups in propensity-score matched samples Stat Med 2009 28 25 3083 3107 10.1002/sim.3697 PMC3472075 19757444

[35] Benjamini Y Hochberg Y Controlling the false discovery rate: a practical and powerful approach to multiple testing J R Stat Soc Series B Stat Methodol 1995 57 01 289 300

[36] Rothman K J No adjustments are needed for multiple comparisons Epidemiology 1990 1 01 43 46 2081237

[37] Bart Y Chauhan S P Fishel Bartal M Blackwell S Sibai B M Equivalence of single and standard doses of antenatal corticosteroids for late preterm neonatal outcomes: insights from a secondary analysis Am J Obstet Gynecol 2024 231 06 6450 6.45E9 10.1016/j.ajog.2024.04.004 38588964

[38] Reddy U M Deshmukh U Dude A Harper L Osmundson S S Society for Maternal-Fetal Medicine (SMFM). Society for Maternal-Fetal Medicine Consult Series #58: Use of antenatal corticosteroids for individuals at risk for late preterm delivery: Replaces SMFM Statement #4, Implementation of the use of antenatal corticosteroids in the late preterm birth period in women at risk for preterm delivery, August 2016 Am J Obstet Gynecol 2021 225 05 B36 B42 10.1016/j.ajog.2021.07.023 34363784

[39] World Health Organization WHO Recommendations on Antenatal Corticosteroids for Improving Preterm Birth Outcomes Geneva World Health Organization 2022 36279381

[40] Gyamfi-Bannerman C Clifton R G Tita A TN Eunice Kennedy Shriver Maternal-Fetal Medicine Units Network Neurodevelopmental outcomes after late preterm antenatal corticosteroids: the ALPS follow-up study JAMA 2024 331 19 1629 1637 10.1001/jama.2024.4303 PMC11044009 38656759

[41] Aviram A Murphy K McDonald S Antenatal corticosteroids and neurodevelopmental outcomes in late preterm births Arch Dis Child Fetal Neonatal Ed 2022 107 03 250 255 10.1136/archdischild-2021-322152 34588179

[42] Liauw J Campbell K SJ Foggin H Antenatal corticosteroids and child neurodevelopment: a systematic review and meta-analysis Obstet Gynecol 2025 146 03 360 376 10.1097/AOG.0000000000005950 PMC12363325 40472380

[43] Clapp M A Li S Cohen J L Betamethasone exposure and neonatal respiratory morbidity among late preterm births by planned mode of delivery and gestational age Obstet Gynecol 2024 144 06 747 754 10.1097/AOG.0000000000005756 39388700

[44] Thornton P S Stanley C A De León D D Pediatric Endocrine Society Recommendations from the Pediatric Endocrine Society for evaluation and management of persistent hypoglycemia in neonates, infants, and children J Pediatr 2015 167 02 238 245 10.1016/j.jpeds.2015.03.057 PMC11891912 25957977

