# Supplementary material for: Antenatal Corticosteroid Administration in Late Preterm Singleton Pregnancies: A Propensity Score-weighted Analysis of Neonatal Outcomes
Source: Am J Perinatol. 2026 May 18;43(15):2081–90. doi: 10.1055/a-2869-5471 (PMC13709877; doi:10.1055/a-2869-5471)
Supplement: Supplementary file 1 [file 10-1055-a-2869-5471_29590713.pdf]

SUPPLEMENTARY MATERIALS

Antenatal Corticosteroid Administration in Late Preterm Singleton Pregnancies: A Propensity Score-Weighted Analysis of Neonatal Outcomes

Contents:

- Supplementary Methods
- Supplementary Table S1. ACS Dose and ACS-to-Delivery Timing Among Exposed Pregnancies
- Supplementary Table S2 Initial Respiratory Support Modality by Exposure Group
- Supplementary Table S3 Continuous Neonatal Outcomes (Unweighted Group Summaries; IPTW Mean Differences)
- Supplementary Table S4 Sensitivity Analyses for the Primary Outcome (Initial Respiratory Support)
- Supplementary Figure S1 Propensity score distribution by exposure
- Supplementary Figure S2 Stabilized IPTW distribution (trimmed)

SUPPLEMENTARY METHODS

The source dataset included 1,091 deliveries (2017–2022). Late preterm singleton deliveries were defined as 34+0 to 36+6 weeks (n=1,087). For the primary analytic cohort, pregnancies receiving antenatal corticosteroids before 34 weeks (n=75) were excluded to reduce exposure mixing, yielding 1,012 deliveries (126 exposed; 886 unexposed).

Propensity scores were estimated using logistic regression with prespecified covariates: maternal age, body mass index, gravidity, parity, conception method, maternal comorbidity group, medication use group, maternal infection status, indication for delivery, delivery mode, and fetal sex. Stabilized inverse probability of treatment weights were trimmed at the 1st and 99th percentiles. Propensity score overlap and the trimmed weight distribution are shown in Figures S1 and S2, respectively.

Covariate balance was assessed using absolute standardized mean differences (SMD) before and after weighting. An SMD <0.10 was considered indicative of acceptable balance. After weighting, all covariates achieved SMD <0.10, indicating adequate balance in the weighted pseudo-population (see Figure 2 in the main manuscript).

For the doubly robust sensitivity analysis, weighted logistic regression models additionally included all propensity score covariates as regressors. This approach provides a second chance for correct specification: the treatment effect estimate is consistent if either the propensity score model or the outcome model is correctly specified. For the sensitivity cohort analysis, pregnancies that received ACS before 34 weeks (n=75) were re-included, and early ACS exposure was added as an additional covariate in both the propensity score and outcome models. An additional sensitivity analysis excluded delivery mode from the propensity score to evaluate the impact of measured delivery-mode adjustment on the primary estimate.

The source dataset encoded late preterm ACS dose and ACS-to-delivery timing for exposed pregnancies; these variables were summarized descriptively because complete-course and latency-specific analyses were limited by small exposed-cell sizes. Labor induction, total hospital length of stay, antibiotic administration, active versus expectant management of premature rupture of membranes, planned versus intrapartum cesarean delivery, and labor status were not reliably available in the retrospective dataset and therefore could not be reported or modeled.

Supplementary Table S1 ACS Dose and ACS-to-Delivery Timing Among Exposed Pregnancies

Characteristic	n	%
**Documented ACS dose**		
Single documented dose	108	85.7
Double documented dose	18	14.3
**ACS-to-delivery interval**		
0–6 hours	30	23.8
6–24 hours	40	31.7
24–48 hours	23	18.3
2–7 days	25	19.8
>7 days	8	6.3

Abbreviations: ACS, antenatal corticosteroids. One record coded as "6" was grouped with the 0–6 hour category. Percentages use the 126 late preterm ACS-exposed pregnancies as the denominator.

Supplementary Table S2 Initial Respiratory Support Modality by Exposure Group

Initial support modality	ACS (n = 126)	No ACS (n = 886)
No support	83 (65.9)	663 (74.8)
Hood oxygen only	14 (11.1)	34 (3.8)
CPAP	21 (16.7)	162 (18.3)
Positive-pressure ventilation (PPV)	6 (4.8)	22 (2.5)
Intubation	2 (1.6)	5 (0.6)
Any initial respiratory support	43 (34.1)	223 (25.2)
Higher-intensity support (CPAP/intubation)	29 (23.0)	189 (21.3)

Abbreviations: ACS, antenatal corticosteroids; CPAP, continuous positive airway pressure; PPV, positive-pressure ventilation (coded as PBV in the source dataset). Values are n (%). Higher-

intensity support was defined descriptively as CPAP, positive-pressure ventilation (PPV; coded as PBV in the source dataset), or intubation.

Supplementary Table S3 Continuous Neonatal Outcomes (Unweighted Group Summaries; IPTW Mean Differences)

Outcome	ACS mean (SD)	No ACS mean (SD)	Crude MD (95% CI)	IPTW MD (95% CI)	P
NICU length of stay, days	3.01 (8.63)	1.72 (6.31)	1.28 (−0.27 to 2.84)	0.83 (−0.49 to 2.15)	.218
Oxygen days total	1.73 (6.90)	0.75 (2.78)	0.98 (−0.23 to 2.20)	0.95 (−0.09 to 1.99)	.074
Invasive MV days	0.50 (3.14)	0.10 (0.78)	0.40 (−0.15 to 0.95)	0.37 (−0.21 to 0.95)	.213
Nasal/non-invasive ventilation days	0.48 (2.13)	0.28 (0.97)	0.21 (−0.17 to 0.58)	0.21 (−0.12 to 0.53)	.221
Apgar score at 1 minute	7.19 (1.75)	7.41 (1.53)	−0.22 (−0.54 to 0.10)	−0.09 (−0.46 to 0.29)	.652
Apgar score at 5 minutes	8.96 (1.22)	8.98 (1.33)	−0.02 (−0.24 to 0.21)	0.03 (−0.24 to 0.31)	.806

Abbreviations: ACS, antenatal corticosteroids; CI, confidence interval; IPTW, inverse probability of treatment weighting; MD, mean difference; MV, mechanical ventilation; NICU, neonatal intensive care unit; SD, standard deviation.

P-values are from IPTW-weighted linear regression with robust standard errors.

Supplementary Table S4 Sensitivity Analyses for the Primary Outcome (Initial Respiratory Support)

**Analysis method**	**n**	**OR**	**95% CI**	**P**
Unadjusted	1,012	1.54	1.03–2.29	.034
Adjusted	1,012	1.34	0.87–2.06	.184
IPTW (primary)	1,012	1.40	0.90–2.18	.130
IPTW + DR	1,012	1.41	0.87–2.27	.159
Include early ACS (IPTW)	1,087	1.37	0.89–2.09	.150
Include early ACS (IPTW + DR)	1,087	1.40	0.89–2.21	.148
Exclude delivery mode from PS (IPTW)	1,012	1.54	1.01–2.35	.046
Exclude delivery mode from PS + DR	1,012	1.57	1.02–2.42	.042

Abbreviations: ACS, antenatal corticosteroids; CI, confidence interval; DR, doubly robust; IPTW, inverse probability of treatment weighting; OR, odds ratio.

The primary outcome was documented initial respiratory support at birth/early neonatal stabilization. The main cohort excludes pregnancies with ACS before 34 weeks; the sensitivity cohort includes these pregnancies and adjusts for early ACS exposure. The delivery-mode sensitivity analysis excludes measured delivery mode from the propensity score but does not resolve residual confounding from planned versus intrapartum cesarean delivery or absence of labor.

SUPPLEMENTARY FIGURES

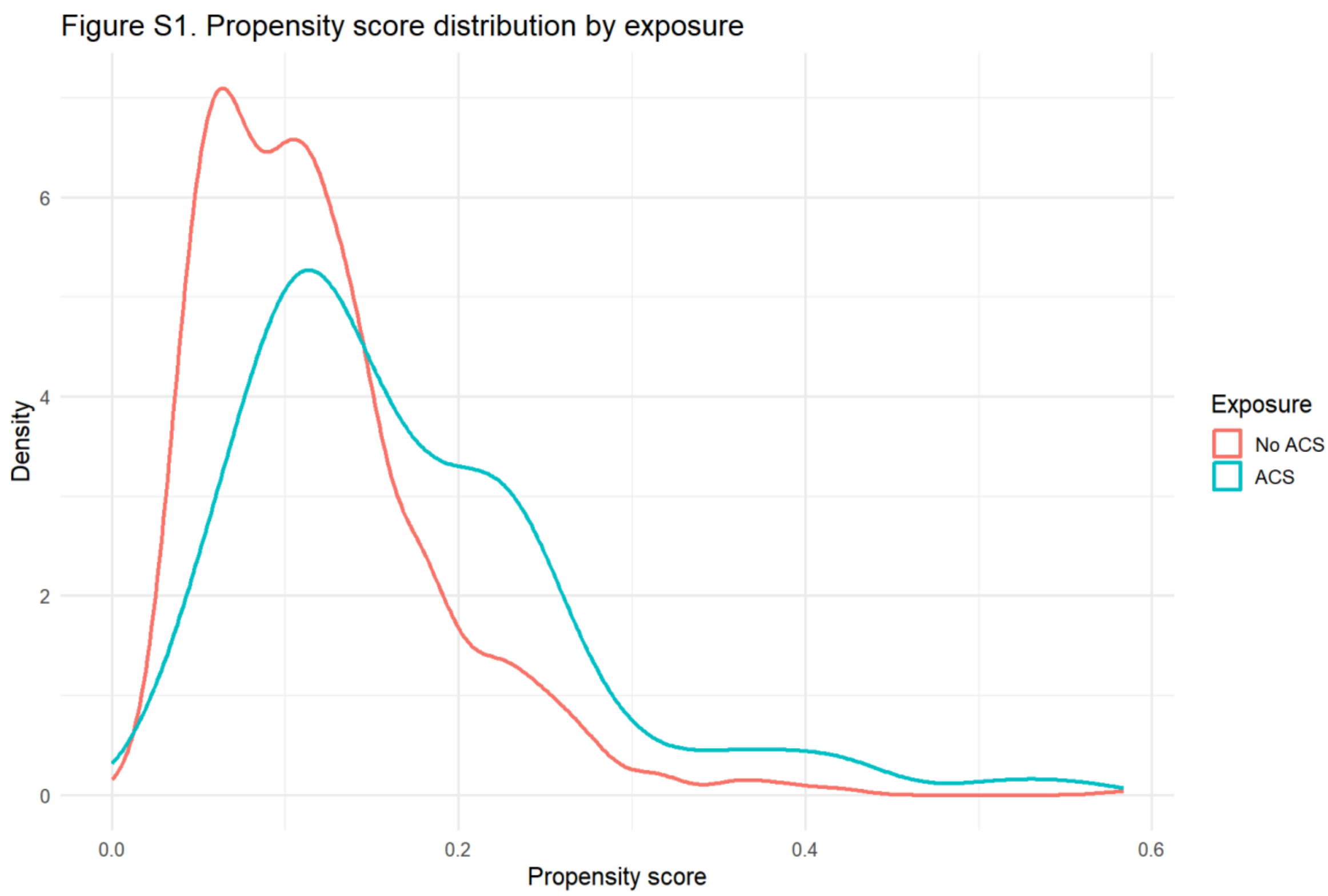

Supplementary Fig. S1 Propensity score distribution by exposure. Kernel density plot of estimated propensity scores for antenatal corticosteroid (ACS) exposure in the primary analytic cohort (n=1,012). The distributions show adequate overlap between exposed and unexposed groups, supporting the use of inverse probability of treatment weighting.

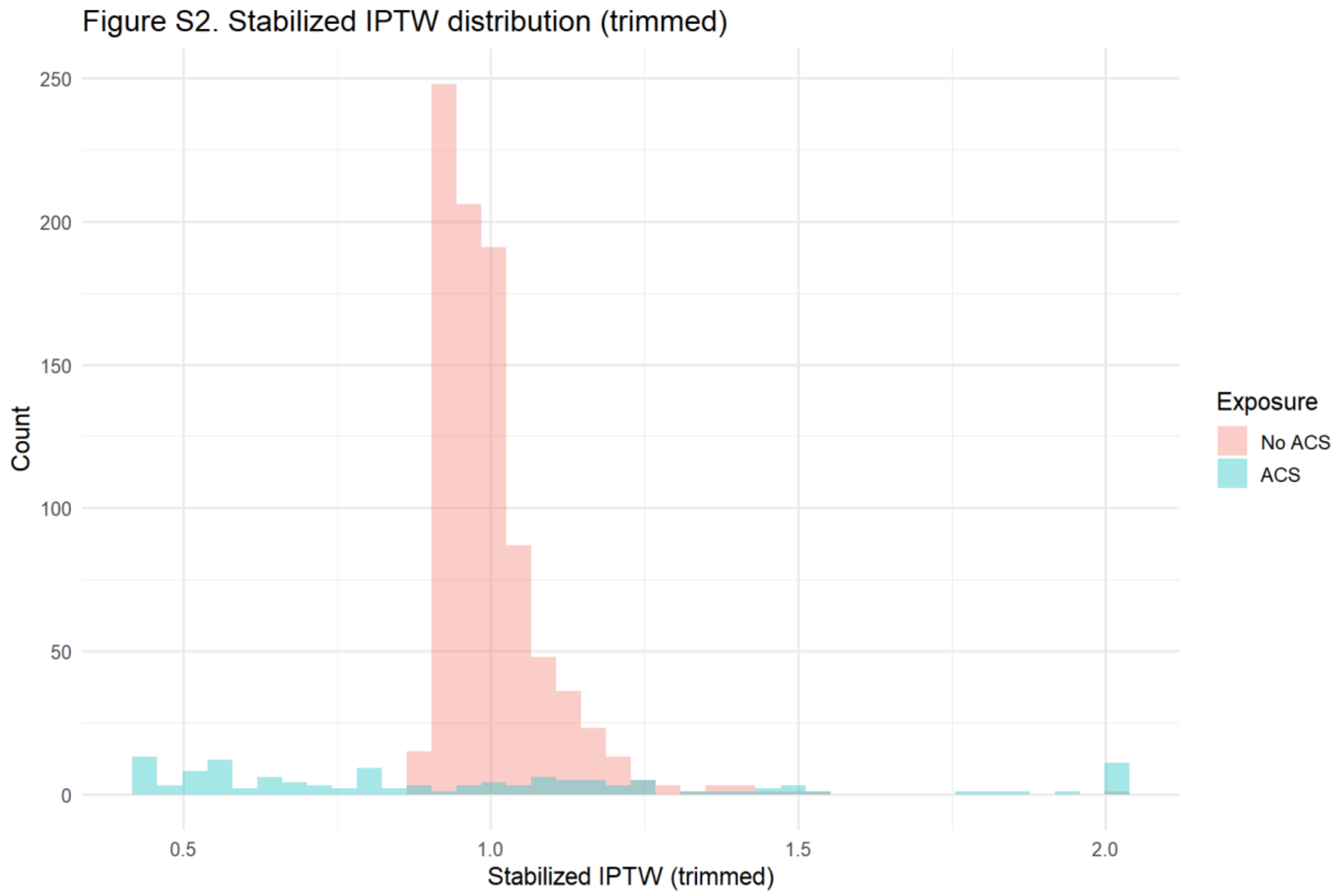

Supplementary Fig. S2 Stabilized IPTW distribution (trimmed). Histogram of stabilized inverse probability of treatment weights after trimming at the 1st and 99th percentiles. The majority of weights are concentrated near 1.0, indicating reasonable weight stability and minimal influence of extreme weights on the treatment effect estimates.
